# Allopurinol desensitization with A 2 weeks modified protocol in an elderly patients with multiple comorbidities: a case report

**DOI:** 10.1186/1710-1492-10-52

**Published:** 2014-10-22

**Authors:** Adile Berna Dursun, Osman Z Sahin

**Affiliations:** Department of Internal Medicine, Division of Immunology and Allergic Diseases, Recep Tayyip Erdogan University, School of Medicine, Rize, Turkey; Department of Internal Medicine, Division of Nephrology, Recep Tayyip Erdogan University, School of Medicine, Rize, Turkey

**Keywords:** Allopurinol hypersensitivty, Oral drug desensitization, Slow desensitization, Hyperuricemia, Gout, Maculopapular exanthema

## Abstract

**Background:**

Allopurinol is an effective urate-lowering drug that is well tolerated by the majority of patients. Patients with chronic renal insufficiency have an increased risk of hypersensitivity reactions with allopurinol.

**Case presentation:**

75 year old male patient with gout, renal insufficiency, history of metastatic colorectal carcinoma status post-resection was referred to Allergy clinic for a maculopapular eruption that developed 1 week after initiating therapy with allopurinol. The rash resolved with discontinuation of allopurinol. However, his serum urate level rose to 19.9 mg/dl. We initially proposed a slow 4 week oral allopurinol desensitization. The treating nephrologist felt it was critical to lower urate more rapidly. As a result, we modified the dose and standard 4 week protocol down to 2 weeks. A suspension of allopurinol was prepared by the allergy nurse practitioner with a 300 mg allopurinol tablet. The sensitization protocol was modified as a starting dose of 0.3 mg escalating to a final dose of 300 mg/day in 2 weeks. There was no reaction during or after the desensitization. The patient’s urate level normalized (6.3 mg/dl) and has continued on 300 mg allopurinol daily without reaction.

**Conclusion:**

A 2 week modified allopurinol desensitization protocol is a safe alternative for elderly patients with multiple comorbidities.

Allopurinol is a first line drug for treatment of gout and works by inhibition of xanthine oxidase leading to decreased uric acid produciton [1]. Although it is a well-tolerated drug, approximately 2% of patients treated with allopurinol have adverse reactions such as fixed drug eruptions (FDE), pruritic maculopapular exanthema (MPE) and minor vasculitis whicch resolve with cessation of the drug which limits it use in these individuals [2]. Life-threatening reactions such as DRESS; SJS or TEN, occur very infrequently (0.2%) [3, 4].

Approach to allopurinol hypersensitivity firstly includes withdrawing allopurinol, giving supportive measures such as modifying diet and alcohol intake and corticosteroids in severe cases [3]. The other uricosuric drugs (probenicid, benzbromarone and sulfinpyrazone) are another option in the chronic treatment of gout [5]. When allopurinol treatment is necessary and there is no alternative drugs in the local market, drug desensitization should be considered except for severe hypersentivity reactions [4].

After first publication of desensitization with allopurinol in 1976, several case reports with oral and intravenous protocols were published [5, 6]. Slow drug desensitization protocol lasting in 28-81 days are recommended particularly for elderly patients with multiple comorbidities [2]. We present a successful modified 2 weeks oral allopurinol desensitization in an elderly patient with multiple comorbidities.

## Case presentation

A 75 years old male was consulted due to hypersensitivty reaction to allopurinol. He had chronic renal disease, metastatic colorectal carcinoma status post-resection. Neither the patient nor his family members had a history of drug hypersensitivity. A week after starting allopurinol (300 mg/day), he presented to dermatology out-patient clinic with rashes on his lower extremities. He was diagnosed with allopurinol-induced MPE. Allopurinol was discontinued and he was placed on 5 days of oral corticosteroids. His rash resolved however, his serum urate level rose to 19.9 mg/dl (normal range 3-7.5 mg/dl) and he experienced a gout attack. There were no alternative urate lowering drugs in our market. Febuxostat could be obtained with board report from Ministry of Health but would take at least 3 weeks.

Due to his critic condition, a 28-days allopurinol desensitization protocol was modified to 2 weeks and was performed without premedication (Table 1). The protocol was modified from a protocol developed by Gomes et al. [5]. The soluble suspension of allopurinol was prepared by allergy nurse practitioner as described in Table 2. Solutions were well tolerated by the patient without complaints. The patient was seen at the out-patient clinic on 1^st^, 6t^h^, 9^th^, 12^th^ and 16^th^ days of the protocol. No reaction occured during the desensitization or during continued daily dosing of allopurinol 300 mg. The uric acid level dropped to 6.3 mg/dl with therapy.

**Table 1:** **2 weeks allopurinol desensitization protocol**

DAY	DRUG (Allopurinol)	DOSE (ml)	DOSE (mg)
1	Solution A, 0.3 mg/ml	1	0.3
2		2	0.6
3		4	1.2
4		8	2.4
5		10	3
6	Solution B, 6 mg/ml	3	18
7		6	36
8		10	60
9	Allopurinol tablet (100-300 mg)		75
10			100
11			125
12			150
13			175
14			225
15			250
16			300

**Table 2:** **Description of preparing allopurinol for desensitization**

Source solution	150 mg allopurinol tablet + 50 ml 5% dextrose = 3 mg/ml
Solution A	1:10 source solution (0.3 mg/ml)
Solution B	300 mg allopurinol tablet + 50 ml 5% dextrose = 6 mg/ml

## Discussion

Although allopurinol hypersensitivity is relatively uncommon, up to 2% of patients who use this drug develop hypersensitivity reactions such as FDE, pruritic MPE and vasculitis [2]. Despite the fact that new drugs have been developed to treat hyperuricemia, practical therapeutic choices remain limited. Febuxostat is a good option for patients with allopurinol hypersensitivity, but its accessibility may be very limited in some countries [2, 4]. In this situation, desensitization procedure should be considered, except for patients with severe hypersensitivity to allopurinol. FDE and maculopapular exanthema is an appropriate indication for desensitization with allopurinol [5].

The pathophysiology underlying this hypersensitivity reaction remains unknown, but several risk factors such as the presence of the HLA-B58.01 allele, the dose taken and renal failure have been proposed. Recently Yun et al. found that allopurinol allergic patients are primarily sensitized to oxypurinol. Despite the prevailing dogma that type B adverse drug reactions are dose independent, they concluded that allopurinol hypersensitivity is primarily driven by oxypurinol-specific T cell response in a dose-dependent manner, particular in the presence of HLA-B58.01 allele [7].

Different mechanisms, including hapten inhibition, mast cell and basophil mediator depletion, IgE consumption and mast cell desensitization, have also been proposed to explain the temporary immunological tolerance induced by a desensitization protocol to a drug. The mechanism of slow desensitization is more obscure. It has been suggested that gradually increasing antigen doses allows metabolic adaptation resulting in increased clearance of reactive drug metabolites [2].

There are several oral slow desensitization protocols lasted in 28 to 81 days in the literature [5, 8–10]. Time to reach therapeutic doses is longer particularly in elderly patients with multiple comorbidities for safety issues [2]. However if there is clinical urgency, new or modified protocols could be tailored. The original protocol was generated by Umperierrez et al. [9] for a patient with FDE to allopurinol, then Gomes et al. adapted the protocol for also systemic reactions to allopurinol [5]. In the Gomes study, the type of reaction was FDE in three patients, urticarial with or without angioedema in two, anaphylaxis in one and pruritic MPE in one patient. They reported 100% overall success rate of desensitization with no complications in the progression of the protocol in three patients (43%) and the remaining had mild to moderate skin reactions [5]. Even though the protocol was initially created for desensitization of patients with local skin reactions, Gomes et al. confirmed that it is also effective in desensitization of patients with systemic reactions [5]. Thus we also modified the same protocol for patient with MPE to allopurinol and the successful outcome revealed the flexibility of slow desensitization protocols.

It is important to share new or modified drug desensitization protocols, whether they have success or not, in order to build up experiences in a field of insufficient data [11]. We present a successful shortened oral desensitization protocol for allopurinol hypersensitivity. The presented case demonstrated that a 2 weeks oral allopurinol desensitization protocol can be safely undertaken in elderly patients with multiple comorbidities.

## Consent

Written informed consent was obtained from the patient for publication of this case report and any accompanying images. A copy of written inform consent is available for review by the Editor-in-Chief of this journal.
